# Bibliometric analysis of cognitive dysfunction after traumatic brain injury

**DOI:** 10.3389/fnins.2025.1534735

**Published:** 2025-02-25

**Authors:** Jihua Hu, Ruiting Zhu, Xin Zhang, Yuchen Zhang, Jixin Liu, Wenyang Wang, Chiyin Li, Tong Yang, Ming Zhang, Xuan Niu

**Affiliations:** ^1^Department of Medical Imaging, The First Affiliated Hospital of Xi’an Jiaotong University, Xi’an, China; ^2^School of Future Technology, Xi'an Jiaotong University, Xi’an, China; ^3^Department of Pharmacy, Xi’an Honghui Hospital, Xi’an, China; ^4^Department of Nuclear Medicine, The First Affiliated Hospital of Xi’an Jiaotong University, Xi’an, China; ^5^Xi’an Key Laboratory of Intelligent Sensing and Regulation of Trans-Scale Life Information, School of Life Science and Technology, Xidian University, Xi’an, China; ^6^Department of Rehabilitation Medicine, The First Affiliated Hospital of Xi’an Jiaotong University, Xi’an, China

**Keywords:** traumatic brain injury, head injury, cognitive dysfunction, cognition, bibliometric analysis

## Abstract

**Background:**

Cognitive dysfunction after traumatic brain injury (TBI) significantly reduces quality of life and imposes a heavy burden on society. A detailed examination of research trends of cognitive dysfunction following TBI has not yet been conducted. This study aimed to examine the bibliometric analysis of cognitive dysfunction after traumatic brain injury over the past 20 years.

**Methods:**

Literature on bibliometric analysis was retrieved from the Web of Science Core Collection (WoSCC) and Science Citation Index Expanded (SCI-E) from 2004 to 2023. The type of literature and the language were refined. A total of 1,902 articles were used for bibliometric analysis, including 1,543 (81.1%) original articles and 359 (18.9%) review articles. Data were retrieved on June 5, 2024.

**Results:**

The publication volume of articles was increasing year by year, with articles published in 537 journals. The *Journal of Neurotrauma*, with 130 articles, was the most productive and influential journal. The University of California System led in the number of articles published. There were 9,002 authors from 62 countries/regions. The USA and China were the top-ranked countries by article count. Pandharipande PP authored the highly cited article. Pick CG, as the author with the highest h-index. The top three of author keywords were traumatic brain injury, cognitive impairment, and mild traumatic brain injury. The topics of cognitive dysfunction after TBI were ferroptosis, cognitive decline, spinal cord injury, and prognosis.

**Conclusion:**

Our findings provide valuable insights into cognitive dysfunction following TBI and highlight emerging trends for future research.

## 1 Introduction

Traumatic brain injury (TBI) continues to be a global health issue (Maas et al., 2017; Maas et al., 2022; Ye et al., 2024). Cognitive dysfunction is a common negative outcome of TBI (Peters and Lyketsos, 2023; Johnson et al., 2013; Lai et al., 2022). Two weeks after TBI, different qualitative profiles of symptoms and cognitive functioning were found in patients presenting to U.S. level-1 trauma centers (Brett et al., 2021). There was extensive evidence that chronic cognitive problems after TBI result from diffuse axonal injury and widespread disruption of brain connectivity (Jolly et al., 2020). The severity of TBI was linked to cognitive impairments, which can persist for many years after injury (Pandharipande et al., 2013; Draper and Ponsford, 2008; Lu et al., 2023).

Cognitive dysfunction, especially memory impairment, is a typical clinical feature of long-term symptoms caused by repetitive mild TBI (Miyata et al., 2024; Lu et al., 2024). Studies have demonstrated a connection between white matter abnormalities and the severity of current cognitive dysfunction, as well as the extent of cortical amyloid buildup years after moderate-to-severe TBI (Mohamed et al., 2021). These findings suggested a strong association between TBI and cognitive dysfunction.

Although the research on cognitive dysfunction after TBI has drawn considerable attention, there has been little examination of research trends in this field. Bibliometric analysis can provide statistical descriptions of published literature. However, bibliometric research related to cognitive dysfunction after TBI has not been extensively published. To address this knowledge gap, we conducted a bibliometric analysis to examine research trends of cognitive dysfunction after TBI.

## 2 Materials and methods

### 2.1 Data sources

The data for this study were obtained from the WoSCC. In order to guarantee the precision and excellence of retrieval, the Citation Index was configured to SCI-Expanded (SCI-E). Time span was from January 1, 2004 to December 31, 2023. The type of literature was refined as original article or review article, with only English-language publications included. Non-English articles were excluded from the analysis.

### 2.2 Search strategy

To ensure precision, the study utilized the title (TI), abstract (AB), and author keywords (AK) in advanced search (Aria and Cuccurullo, 2017; Ranjbari et al., 2022). The search strategy for this study was defined as follows: (TI = (“cognitive dysfunction”) OR AB = (“cognitive dysfunction”) OR AK = (“cognitive dysfunction”)) AND (TI = (“traumatic brain injury”) OR AB = (“traumatic brain injury”) OR AK = (“traumatic brain injury”)). The literature search was conducted using Medical Subject Headings (MeSH) in PubMed. For traumatic brain injury related studies, we used “Brain Injuries, Traumatic”[MeSH] as the standardized term. For cognitive dysfunction related studies, we used “Cognitive Dysfunction”[MeSH] as the standardized term. The final search strategy consisted of search terms for the concepts of cognitive dysfunction and TBI and these were combined with Boolean logic operators (Supplementary material).

### 2.3 Data standardization

Our data standardization process was systematically conducted using Bibliometrix R. The original bibliometric records were first imported into Biblioshiny and exported to Excel format, maintaining all original data structures. We created two auxiliary files: a synonym. Text file for merging synonymous terms and a remove. Text file for eliminating duplicate/unwanted records. Prior to visualization, we implemented a systematic synonym consolidation strategy by merging uppercase and lowercase variants (e.g., “TBI” and “tbi”), combining abbreviations with their full forms (e.g., “TBI” and “Traumatic Brain Injury”), and standardizing hyphenated and non-hyphenated terms (e.g., “post-traumatic” and “posttraumatic”). The final analysis was performed by simultaneously processing these files with the original download_text file, ensuring data integrity while achieving standardization without direct manipulation of the source data.

### 2.4 Data analysis

The analysis was carried out using Bibliometrix R (Aria and Cuccurullo, 2017) (version 4.3.3) and Microsoft Excel 2021 (Microsoft Corp., Redmond, WA, United States). We conducted three specific types of analyses using Bibliometrix R: (1) Co-authorship network analysis, which revealed international collaboration patterns between countries/regions; (2) Co-citation analysis, which identified influential works through local citations (LC) and total citations (TC); and (3) Keyword trend analysis, which tracked the evolution of research topics from 2004 to 2023, demonstrated through keyword frequency analysis, temporal changes, and thematic maps for two periods. This comprehensive analytical approach using an integrated platform ensured methodological consistency and reproducibility (Ranjbari et al., 2022; Hu et al., 2024). Prior to visualization, we implemented a systematic synonym consolidation strategy by merging uppercase and lowercase variants (e.g., “TBI” and “tbi”), combining abbreviations with their full forms (e.g., “TBI” and “Traumatic Brain Injury”), and standardizing hyphenated and non-hyphenated terms (e.g., “post-traumatic” and “posttraumatic”). Microsoft Excel 2021 was employed for generating the annual scientific output and total citation count.

## 3 Results

### 3.1 Analysis of annual publication distribution

**Figure 1:**
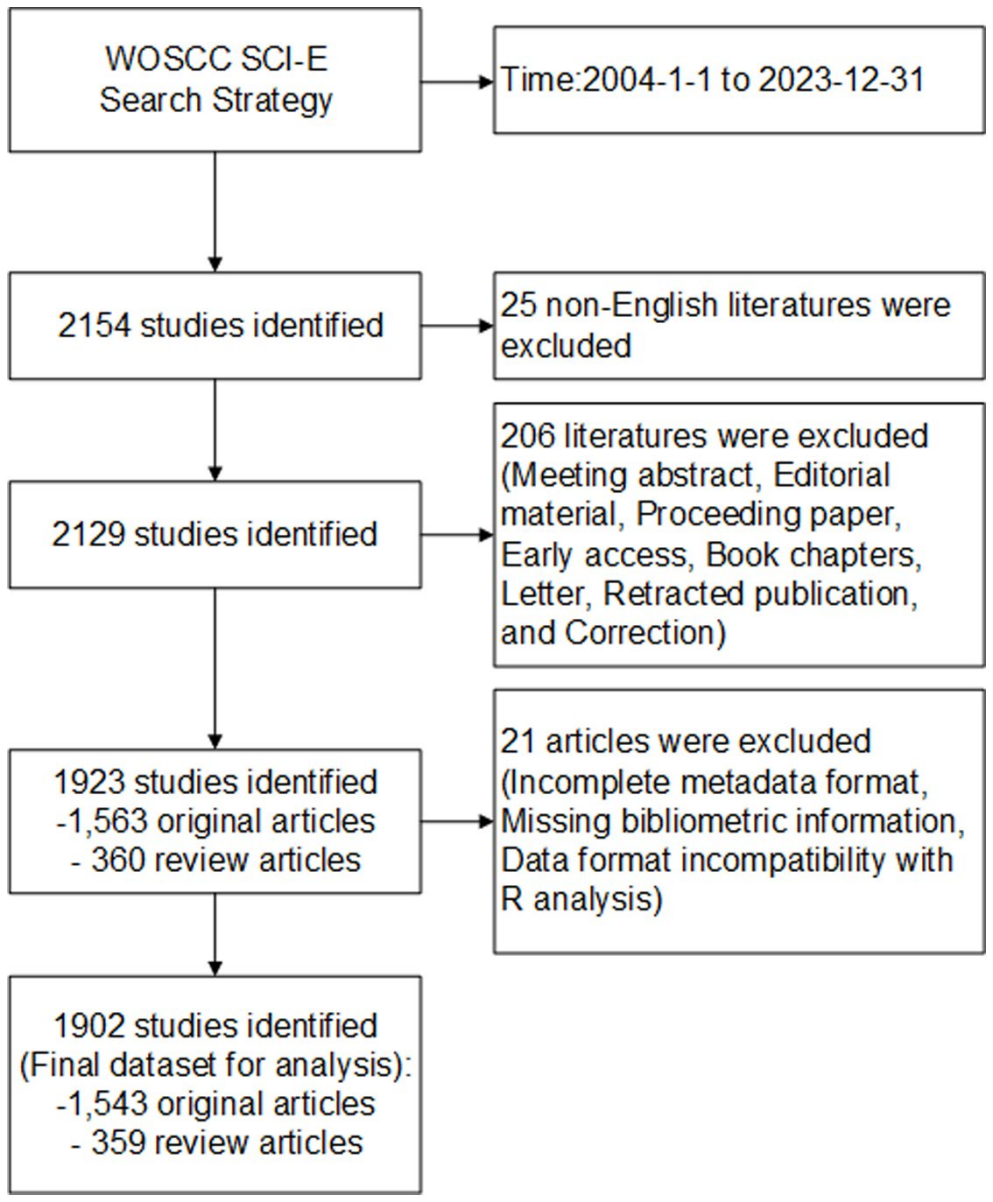
Flow diagram for the screening.

**Figure 2:**
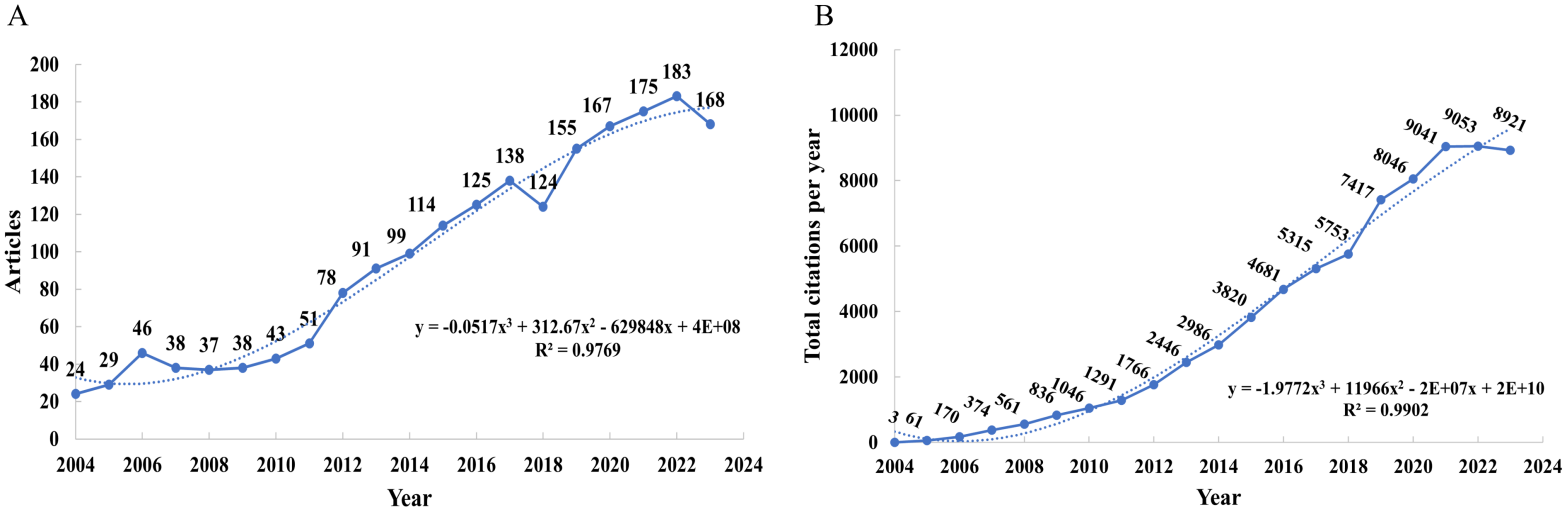
Trends in the number of publications **(A)** and total citations **(B)** growth over time.

From 2004 to 2023, 1,923 publications were identified in WoSCC, containing 1,563 (81.3%) original articles, and 360 (18.7%) review articles (Figure 1). Figure 2A displays the yearly number of publications obtained from the WoSCC. There was a steady increase in the overall amount of published works from 2004 to 2023, showing a rising academic focus on cognitive dysfunction following TBI. Furthermore, a generalized additive model was employed to evaluate the correlation between the quantity of articles and the year of publication.

The annual citation count showed a consistent increase from 2004 to 2023 (Figure 2B). Starting with 3 citations in 2004, the number grew to 1,046 by 2010 and further increased to 4,681 in 2016. Citations continued to rise, reaching 7,417 in 2019 and peaking at 9,053 in 2022, with a slight decrease to 8,921 in 2023. This trend indicates the growing academic influence of research in this field over the past two decades.

### 3.2 The findings of the descriptive bibliometric analysis

#### 3.2.1 Brief bibliographic details

From 2004 to 2023, 1,923 publications were initially identified in WoSCC. During the bibliometric analysis process using Bibliometrix R, 21 articles were automatically excluded from the analysis due to technical limitations, such as incomplete metadata formatting, missing bibliometric information, and data structure incompatibility with R analysis requirements. Table 1 presents the selected dataset, comprising 1,902 articles (1,543 original articles and 359 review articles) published across 537 journals, with an average publication date of 7.35 years. On average, each article received 40.6 citations. The dataset contained a total of 3,758 author keywords and 4,497 keywords plus. There were 9,002 contributors in total, with 53 acting as sole authors and 8,949 as co-authors. Additionally, the annual publications of original and review articles were visualized separately (Supplementary Figure 1), and the results showed that the overall trend of the two publication types remained basically the same, with the trend of review articles changing by 1 year later than that of original articles.

**Table 1:** Key details about the study’s final dataset.

Description	Results
Timespan	2004:2023
Number of journals	537
Number of articles	1902
Annual growth rate %	10.99
Average citations per article	40.6
Average years from publication	7.35
Number of references	79,541
Number of keywords Plus	4,497
Number of author’s Keywords	3,758
Number of Authors	9,002
Authors of single-authored articles	53
Authors of multi-authored articles	8,949
Number of single-authored articles	53
Co-Authors per article	6.36
International co-authorships %	20.35

#### 3.2.2 Core journals

As shown in Table 2, the *Journal of Neurotrauma* demonstrates the highest academic influence in this field, with the highest h-index (45), which is strongly correlated with its leading position in both publication volume (130 papers) and total citations (6,435). Following closely, *Brain Injury* ranks second with an h-index of 31, accompanied by 102 publications and 2,735 citations. This pattern extends across the journal spectrum, indicating a consistent positive correlation between journals’ h-indices and their publication outputs and citation counts. In addition, Supplementary Table 1 shows the top 10 journals in terms of h-index for original articles and review articles, respectively. In the original articles, the most influential journal was *Journal of Neurotrauma*, while in the review articles was *Frontiers in Neurology*. Nine of the top ten journals in original articles coincided with the top 10 journals in total articles, while only four of the review articles overlapped.

**Table 2:** Top 10 most influential journals.

Rank	Journal	h-index	NP	TC
1	Journal of Neurotrauma	45	130	6,435
2	Brain Injury	31	102	2,735
3	Journal of Head Trauma Rehabilitation	22	48	1,288
4	Frontiers in Neurology	21	62	1,284
5	Experimental Neurology	19	29	2,319
6	Journal of Neuroscience	19	22	1928
7	PLoS One	19	34	1,241
8	Journal of the International Neuropsychological Society	17	21	886
9	Clinical Neuropsychologist	16	25	636
10	Brain	15	16	3,257

NP, number of publications; TC, total citations.

**Figure 3:**
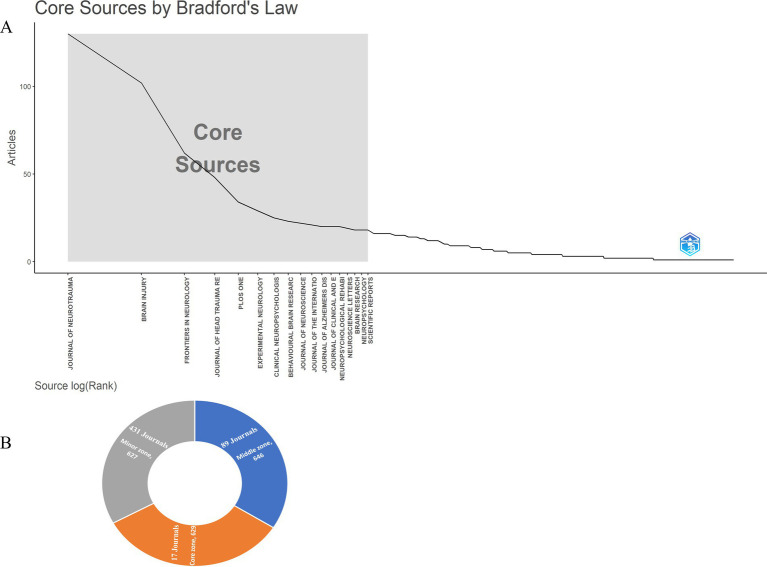
Core journals by Bradford’s law **(A)** and classification of journals according to Bradford’s law **(B)**.

Figure 3 illustrates that the core zone comprised 17 high-quality journals, representing 3.17% of the total, with a total of 629 articles published according to Bradford’s law. Notable journals in this core zone included the *Journal of Neurotrauma*, *Brain Injury*, and *Frontiers in Neurology*. The middle and minor zones contained 646 and 627 articles, respectively, derived from 89 (16.57%) and 431 (80.26%) distinct journals.

#### 3.2.3 Geographic distribution of published articles

**Figure 4:**
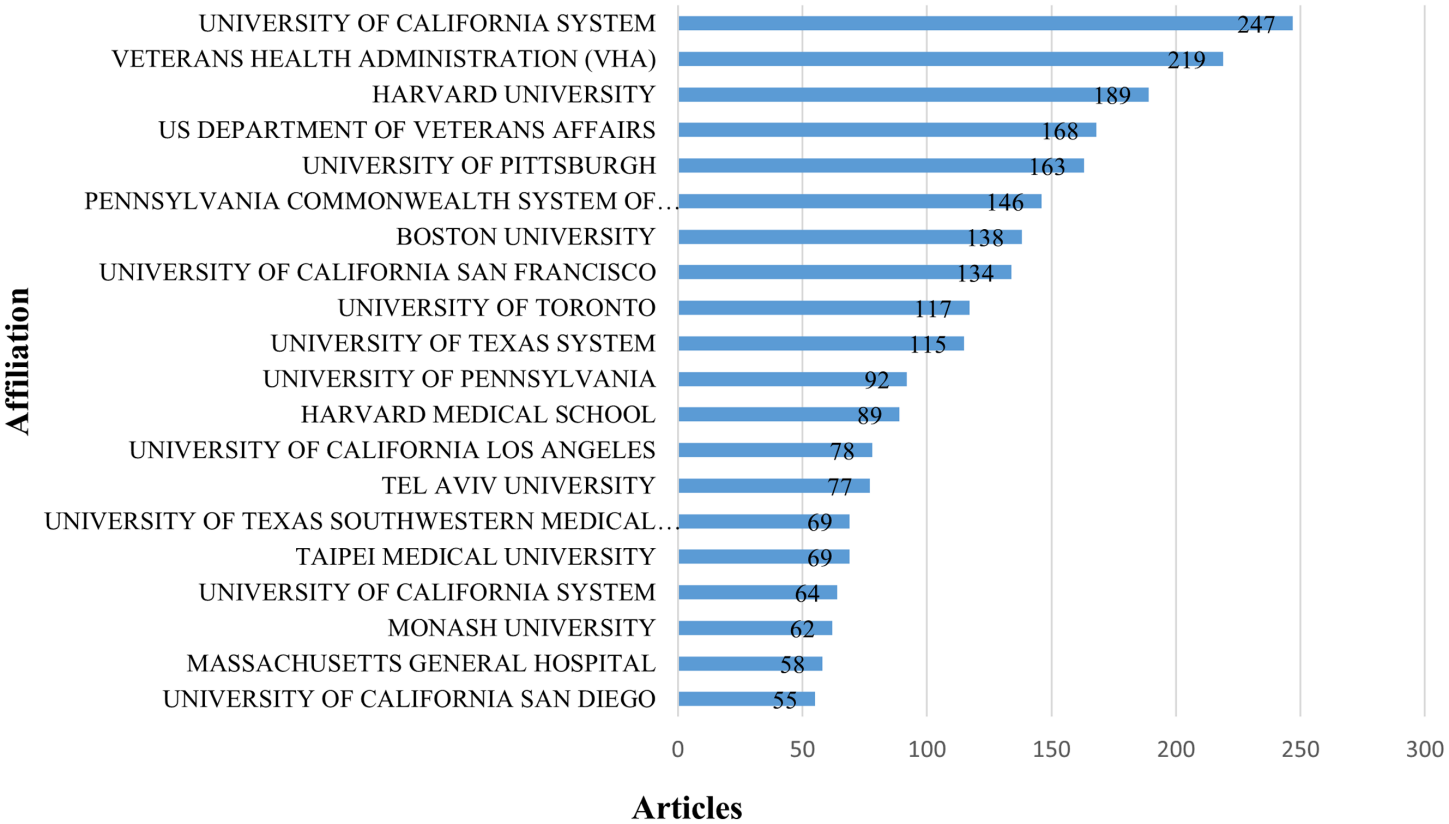
The top 20 most contributing institutions.

The affiliations of all authors involved in the articles were analyzed to assess the collaboration and participation of various countries and regions. The top 20 institutions contributing to research on cognitive dysfunction following TBI from 2004 to 2023 were presented in Figure 4. The University of California System took the top spot with 247 publications, while the Veterans Health Administration (VHA) and Harvard University followed closely behind with 219 and 189 papers, respectively. Moreover, Supplementary Table 2 shows the top 10 institutions in the number of publications of original and review articles. In the original articles, the most productive institution was also the University of California System with 235 publications, while in the review articles was Harvard University with 41 publications. The results showed that nine of the top 10 institutions of original articles coincided with the top 10 institutions of total publications, and six of the top 10 institutions of review articles overlapped.

**Figure 5:**
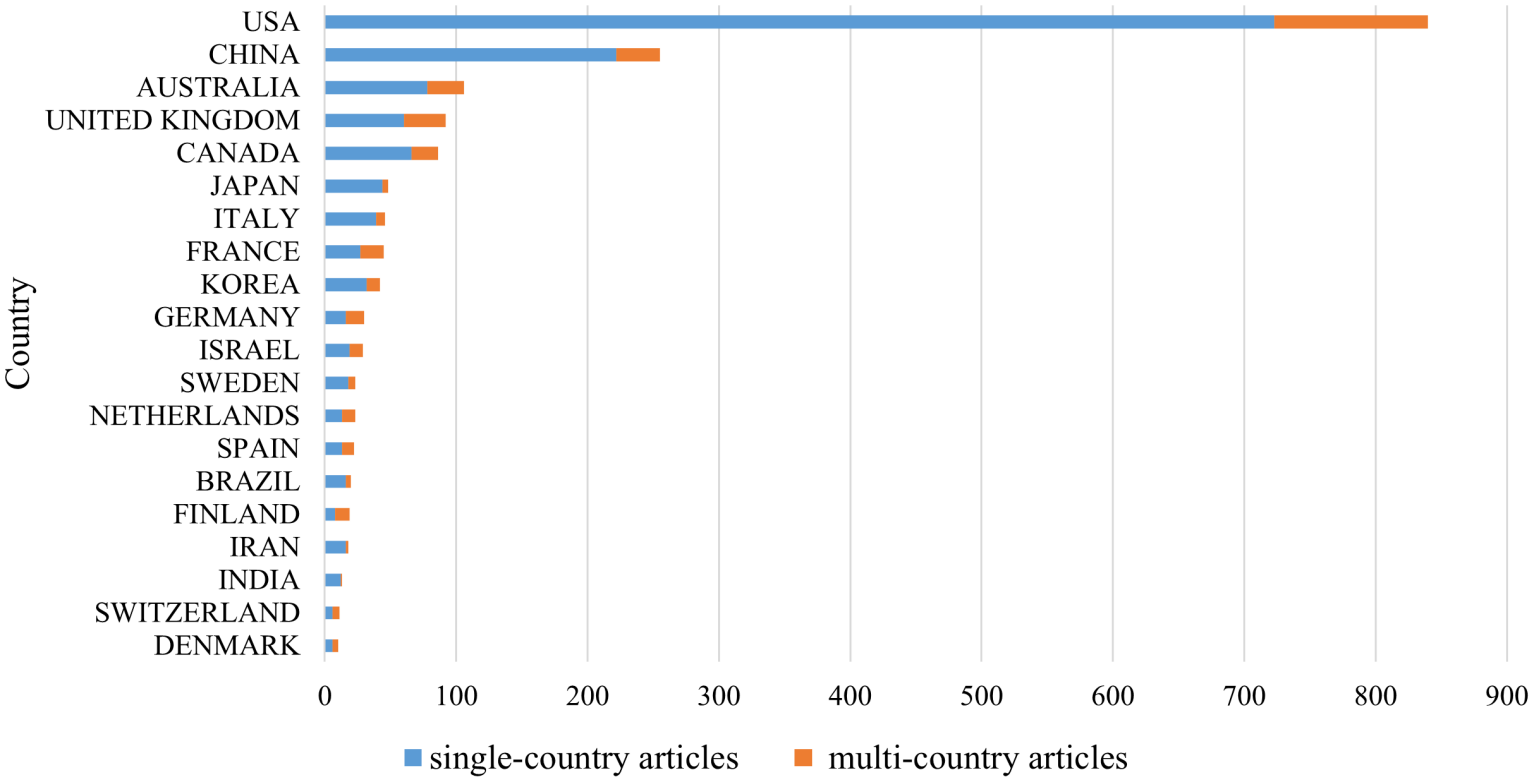
The location of published articles based on the corresponding author's country.

According to the affiliations of the corresponding authors as indicated in the published articles, Table 3 shows a ranking of the top 10 contributing countries/regions. The USA led in terms of publications with 840 articles, followed by China with 255 and Australia with 106. Figure 5 presents the USA also had the highest number of single-country publications (723 articles) and multi-country publications (117 articles). Finland exhibited the highest ratio of multi-country publications, with 8 single-country articles and 11 multi-country articles. Supplementary Table 3 shows the distribution of the top 10 countries by publication output. The United States leads both in original articles (700) and review articles (140), followed by China with 221 and 34 publications, respectively. Notably, Canada ranks third in review articles (27) but fifth in original articles (59).

**Table 3:** Top 10 countries with the highest scientific output.

Rank	Country	Articles
1	USA	840
2	China	255
3	Australia	106
4	United Kingdom	92
5	Canada	86
6	Japan	48
7	Italy	46
8	France	45
9	Korea	42
10	Germany	30

**Figure 6:**
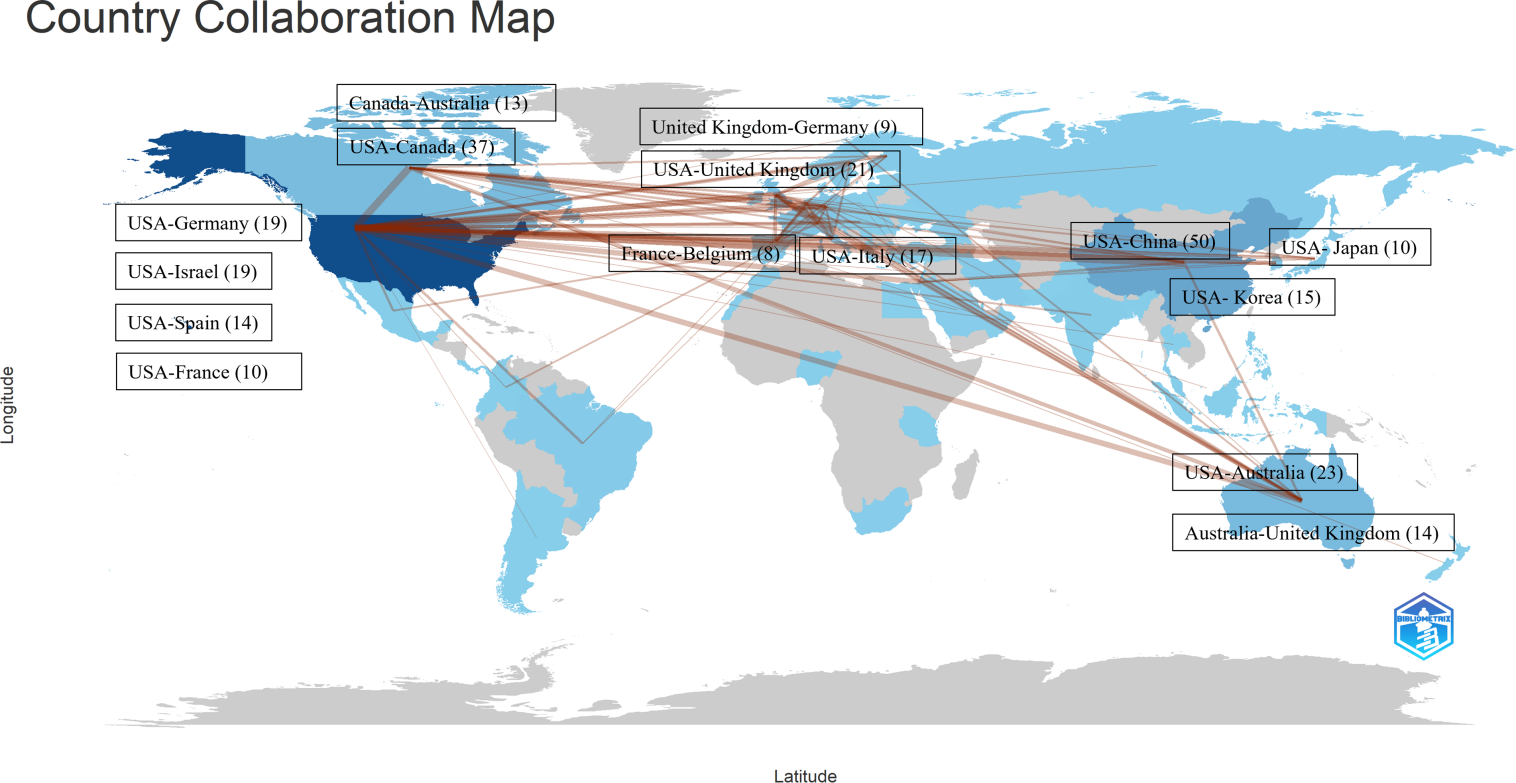
The most frequent co-authorship collaborations among the contributing countries.

Figure 6 demonstrates the co-authorship relationships among the countries/regions contributing to the literature under study. Qualitative analysis of these collaboration networks shows that the USA demonstrates the most extensive international collaboration network, particularly with China (50 collaborations), followed by Canada (37 collaborations). In terms of publication patterns, the USA primarily produces single-country papers (723 papers) with relatively fewer international collaborative papers (117 papers). Notably, Finland, despite its lower total publication count, shows the highest proportion of international collaboration (11 multi-country papers compared to 8 single-country papers). These data reflect different research strategies adopted by different countries in this field: the USA maintains both strong independent research capability and extensive international collaboration networks, while countries like Finland tend to conduct research through international collaboration.

#### 3.2.4 Most influential articles

This study utilized local citation (LC) and total citation (TC) scores to analyze the collected articles. TC represents the overall number of citations received by an article across all databases, whereas LC indicates the citations received from articles within the dataset under study. Normalized citations address the concern that more recent works may not have had sufficient time to accumulate citations compared to earlier works (van Eck and Waltman, 2010).

Tables 4, 5 present the ranking of the top 10 articles within the dataset under examination, as determined by TC, LC, and normalized citations. The article “*Long-term cognitive impairment after critical illness*” had the highest TC value out of the top 10 most cited articles worldwide. The article entitled “*The spectrum of disease in chronic traumatic encephalopathy*” demonstrated the highest LC value. Additionally, the article “*Cognitive functioning ten years following traumatic brain injury and rehabilitation*” exhibited the highest LC/TC ratio. Furthermore, “*Mild Traumatic Brain Injury (mTBI) and chronic cognitive impairment: A scoping review*” was found to be the most cited article based on the normalized LC. The rankings of the top 10 original articles and review articles based on LC value were shown in Supplementary Table 4. Among original articles, “McKee AC, 2013, *Brain*” had the highest LC value of 64; while among review articles, “Smith DH, 2013, *Nature Reviews Neurology*” had the highest LC value of 13.

**Table 4:** Top 10 documents cited globally.

No.	Document	DOI	TC	TC per Year	Normalized TC
1	Pandharipande PP, 2013, New Engl J Med	10.1056/NEJMoa1301372	1,665	138.75	15.53
2	McKee AC, 2013, Brain	10.1093/brain/aws307	1,415	117.92	13.19
3	Johnson VE, 2013, Exp Neurol	10.1016/j.expneurol.2012.01.013	782	65.17	7.29
4	Schliebs R, 2011, Behav Brain Res	10.1016/j.bbr.2010.11.058	767	54.79	7.15
5	Harmon KG, 2013, Brit J Sport Med	10.1136/bjsports-2012-091941	713	59.42	6.65
6	Omalu BI, 2005, Neurosurgery	10.1227/01.NEU.0000163407.92769.ED	698	34.90	7.08
7	Ramlackhansingh AF, 2011, Ann Neurol	10.1002/ana.22455	696	49.71	6.49
8	Gualtieri CT, 2006, Arch Clin Neuropsych	10.1016/j.acn.2006.05.007	583	30.68	5.79
9	Sachdev PS, 2014, Nat Rev. Neurol	10.1038/nrneurol.2014.181	519	47.18	8.60
10	Smith DH, 2013, Nat Rev. Neurol	10.1038/nrneurol.2013.29	502	41.83	4.68

TC, total citations.

**Table 5:** Top 10 documents cited locally.

No.	Document	DOI	LC	TC	LC/TC Ratio (%)	Normalized LC
1	McKee AC, 2013, Brain	10.1093/brain/aws307	106	1,415	7.49	15.36
2	Ramlackhansingh AF, 2011, Ann Neurol	10.1002/ana.22455	60	696	8.62	9.00
3	Smith DH, 2013, Nat Rev. Neurol	10.1038/nrneurol.2013.29	59	502	11.75	8.55
4	Kinnunen KM, 2011, Brain	10.1093/brain/awq347	58	490	11.84	8.70
5	Johnson VE, 2012, Brain Pathol	10.1111/j.1750-3639.2011.00513.x	55	452	12.17	12.36
6	Johnson VE, 2013, Exp Neurol	10.1016/j.expneurol.2012.01.013	55	782	7.03	7.97
7	Omalu BI, 2005, Neurosurgery	10.1227/01.NEU.0000163407.92769.ED	52	698	7.45	8.82
8	Dikmen SS, 2009, J Head Trauma Rehab	10.1097/HTR.0b013e3181c133e9	50	313	15.97	10.38
9	Draper K, 2008, Neuropsychology	10.1037/0894-4105.22.5.618	48	254	18.90	9.20
10	Mcinnes K, 2017, PLoS One	10.1371/journal.pone.0174847	42	287	14.63	17.87

LC, local citations; TC, total citations.

Furthermore, Table 6 presents the top 10 most cited references as determined by citation score. Among these highly referenced articles, “McKee AC, 2013, *Brain*,” “McKee AC, 2009, *Journal of Neuropathology and Experimental Neurology*,” and “Teasdale G, 1974, *Lancet*” were the top three, with 106, 102, and 93 citations, respectively. Notably, “Teasdale G, 1974, *Lancet*,” “McKee AC, 2009, *Journal of Neuropathology and Experimental Neurology*,” and “Folstein MF, 1975, *Journal of Psychiatric Research*” were the top three references in terms of original articles; and for review articles, “McKee AC, 2013, *Brain*,” “McKee AC, 2009, *Journal of Neuropathology and Experimental Neurology*,” and “Moher D, 2015, *Systematic Reviews-London*” were the top three references (Supplementary Table 5).

**Table 6:** Top 10 most local cited references.

NO.	References	DOI	LC
1	McKee AC, 2013, Brain	10.1093/BRAIN/AWS307	106
2	McKee AC, 2009, Journal of Neuropathology and Experimental Neurology	10.1097/NEN.0B013E3181A9D503	102
3	Teasdale G, 1974, Lancet	10.1016/s0140-6736(74)91639-0	93
4	Langlois JA, 2006, Journal of Head Trauma Rehabilitation	10.1097/00001199-200609000-00001	77
5	Plassman BL, 2000, Neurology	10.1212/WNL.55.8.1158	77
6	Folstein MF, 1975, Journal of Psychiatric Research	10.1016/0022-3956(75)90026-6	73
7	Hoge CW, 2008, The New England Journal of Medicine	10.1056/NEJMOA072972	73
8	Rabinowitz AR, 2014, The Psychiatric Clinics of North America	10.1016/J.PSC.2013.11.004	72
9	Fleminger S, 2003, Journal of Neurology, Neurosurgery and Psychiatry	10.1136/JNNP.74.7.857	69
10	Faul MX, 2010, National Center for Injury Prevention and Control.	NA	67

LC, local citations.

#### 3.2.5 Author keywords frequency and trend topics

At the author level, our analysis reveals interesting variations in the relationship between publication metrics and scholarly impact (Table 7). While Pick CG and Sharp DJ both achieved an h-index of 17, their citation patterns differ substantially, with Sharp DJ accumulating significantly more total citations of 3,826 compared to Pick CG with 737 citations. Supplementary Table 6 presents the top 10 most influential authors in cognitive dysfunction after TBI research based on their h-index, number of publications, and TC, categorized by original articles and review articles. In original articles, Pick CG ranked first with an h-index of 16 and 22 publications, while in review articles, Iverson GL led with an h-index of 6 and 7 publications.

**Table 7:** Top 10 most influential authors.

Rank	Author	h-index	NP	TC
1	Pick CG	17	23	737
2	Sharp DJ	17	20	3,826
3	Greig NH	16	18	903
4	Dixon CE	13	13	595
5	Hoffer BJ	13	15	566
6	Rubovitch V	13	15	577
7	Cohen AS	12	13	855
8	McKee AC	12	15	3,463
9	Ponsford J	12	16	663
10	Tweedie D	12	12	726

NP, number of publications; TC, total citations.

**Figure 7:**
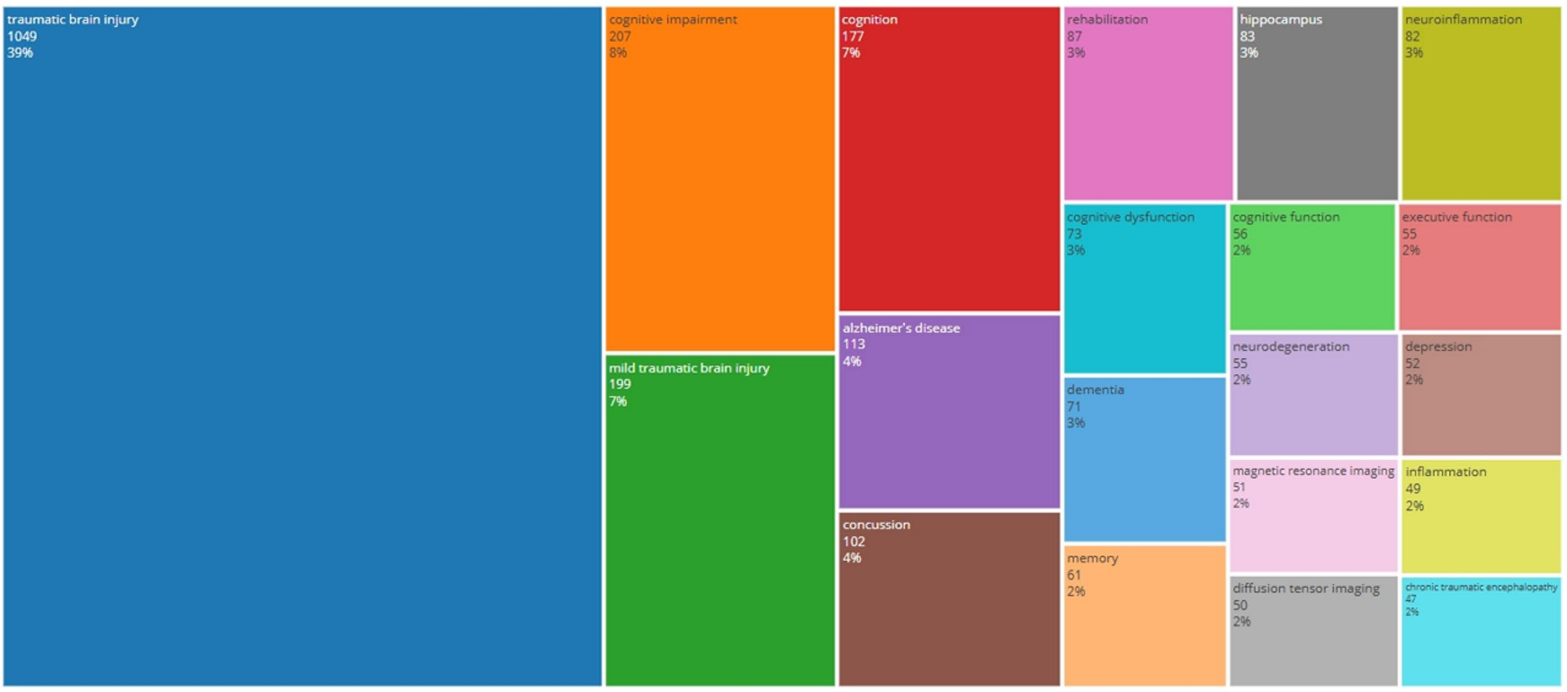
Tree map of the most frequent author keywords.

Analyzing the study areas of cognitive dysfunction following TBI to identify the top 20 author keywords, as shown in Figure 7. The top three author keywords in the dataset were “traumatic brain injury,” “cognitive impairment,” and “mild traumatic brain injury,” with 1,049, 207, and 199 occurrences, accounting for 39, 8, and 7% of all keywords, respectively. Additionally, “cognition” and “Alzheimer’s disease” appeared 177 and 113 times, respectively, accounting for 7 and 4% of the total occurrences. Among the top 20 author keywords identified, “concussion,” “rehabilitation,” and “cognitive dysfunction” were also frequent occurring terms, with 102, 87, and 73 occurrences, respectively, accounting for 4, 3, and 3% of the total terms.

**Figure 8:**
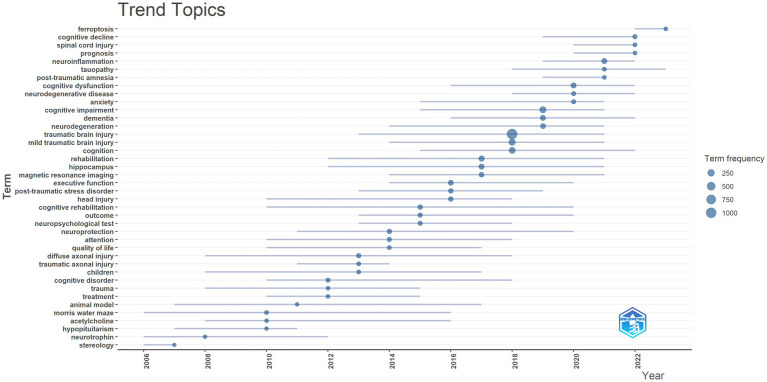
Trend topics in the research domain of cognitive dysfunction after TBI.

The trend topic analysis revealed temporal evolution patterns in the research field (Figure 8). From 2006 to 2022, research themes demonstrated significant dynamic changes. Early research topics such as stereology and neurotrophin emerged during 2006–2008 but gradually diminished thereafter. During the period (2012–2016), several core research directions emerged, including cognitive rehabilitation, neuropsychological test, and attention. Recent research hotspots (2018–2022) primarily focused on ferroptosis, cognitive decline, spinal cord injury, and prognosis. Notably, traumatic brain injury and mild traumatic brain injury showed high term frequencies (500–750 occurrences) and maintained consistent attention throughout the study period. This thematic evolution pattern reflects the field’s progression from basic research toward clinical applications and mechanistic investigations.

#### 3.2.6 Thematic evolution in the field of cognitive dysfunction after TBI

**Figure 9:**
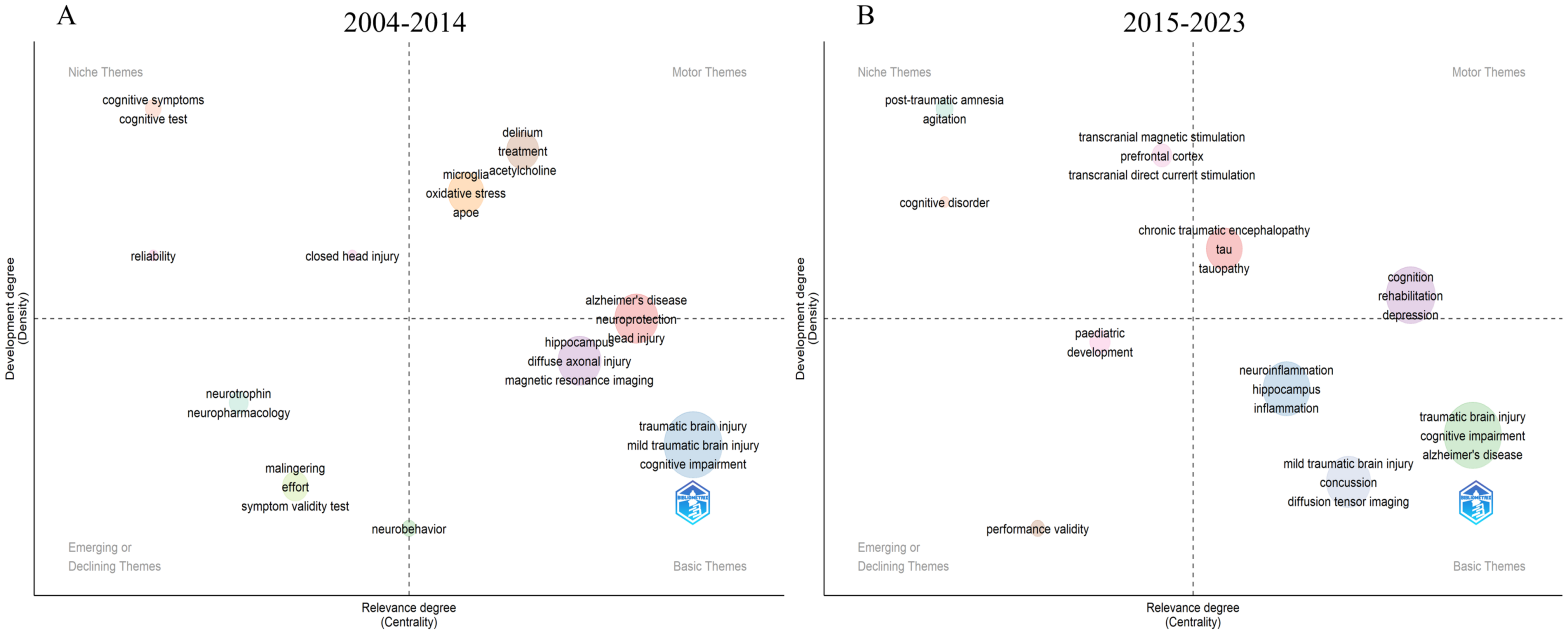
Thematic evolution within the cognitive dysfunction after TBI domain in the periods 2004–2014 **(A)** and 2015–2023 **(B)**.

This study employed a systematic approach to identify emerging trends through thematic evolution analysis. As shown in Figures 9A,B, our dataset was divided into two periods (2004–2014 and 2015–2023), and we analyzed the most frequent keywords using inclusion index weighted by word occurrences. The emerging trends were identified through density-centrality analysis, where density measures the internal strength of theme development, and centrality indicates the importance of theme interactions (Agbo et al., 2021). These thematic maps, based on clusters of keywords, offer a strategic overview of the data. Thematic map for each time period is segmented into four quadrants. Themes positioned in the lower-left quadrant are characterized by lower levels of development and relevance, indicating that these themes are either emerging or in decline (Ranjbari et al., 2022). Conversely, themes situated in the upper-right quadrant exhibit high levels of development and relevance, and are thus referred to as driving or motor themes. The upper-left quadrant delineates specialized themes characterized by low relevance yet high development (niche themes), whereas the lower-right quadrant encompasses fundamental themes that, despite their high relevance, exhibit a low degree of development, thereby forming the foundational themes within the study domain (Ranjbari et al., 2022). Consequently, the density and centrality of the clusters determine the quadrant in which each theme is situated. The research on cognitive dysfunction following TBI identified 11 main themes in its conceptual framework from 2004 to 2014, and 10 main themes from 2015 to 2023.

As shown in Figure 9A, strategic diagram analysis for the period 2004–2014 revealed distinct thematic clusters based on density-centrality metrics. Basic themes with high centrality and density included traumatic brain injury, mild traumatic brain injury, and cognitive impairment, indicating their fundamental role in the field. Motor themes in the high-centrality, high-density quadrant comprised delirium, treatment, and oxidative stress, suggesting their driving force in research development. Niche themes (high density, low centrality) included cognitive symptoms and cognitive tests, while emerging or declining themes (low density, low centrality) encompassed neurotrophin, neuropharmacology, and symptom validity testing.

The 2015–2023 period demonstrated significant thematic evolution (Figure 9B). The basic themes maintained their strong position with traumatic brain injury, cognitive impairment, and Alzheimer’s disease showing high centrality and density. Motor themes evolved to include novel research directions such as transcranial magnetic stimulation, tau pathology, and chronic traumatic encephalopathy. Notably, neuroinflammation and hippocampus-related research emerged in the central region, while post-traumatic amnesia and agitation appeared as niche themes with high internal development but lower external relevance. This strategic distribution reflects the field’s progression toward more specialized therapeutic and mechanistic investigations.

## 4 Discussion

Cognitive dysfunction after TBI has become a research focus. The findings indicated a significant increase in the annual number of publications and citations from 2004 to 2023. Through bibliometric analysis, several key findings emerged, including an examination of literature on cognitive dysfunction following TBI based on citation number, journal, affiliation, country/region, references, author keyword frequency, trend topics, and thematic evolution. Additionally, trend topics related to cognitive dysfunction following TBI were identified, and a thematic evolution spanning the years from 2004 to 2023 in the field of study was delineated.

The analysis of trend topics could reflect the research hotspot iteration in cognitive dysfunction after TBI. The topics over time can be delineated into four distinct phases. Initially, research primarily focused on assessing memory-related cognitive deficits using animal models of TBI (Tayebati, 2006), indicating a growing interest in the cognitive effects of neurological conditions and injuries. The subsequent phase addressed severe brain trauma, such as diffuse axonal injury, which led to cognitive dysfunctions (including memory, attention, and executive function) and psychological impairments (such as post-traumatic stress disorder), significantly impacting patients’ quality of life (Pandharipande et al., 2013; Stocchetti and Zanier, 2016; Vasterling et al., 2012). Consequently, neuroprotection and cognitive rehabilitation for individuals with brain injuries have gained increasing prominence (Draper and Ponsford, 2008; Stocchetti and Zanier, 2016). The third stage witnessed a rapid growth in TBI diagnosis and treatment driven by advanced imaging techniques, with a significant increase in TBI research, particularly in 2018. In this context, neurodegeneration and altered cognitive dysfunction following TBI have emerged as new research topics. Currently, the significance of tauopathy, neuroinflammation, and ferroptosis in the development of cognitive dysfunction post-TBI is increasingly acknowledged (Fang et al., 2023; Xu et al., 2024; Rao et al., 2022; Johnson et al., 2023), highlighting a broader shift toward understanding the biological basis of neurodegeneration and cognitive dysfunction after TBI.

Our finding of trend topics was consistent with the reported studies. Ferroptosis, a form of iron-dependent cell death controlled by lipid oxidation, mainly occurs in the brain (Xu et al., 2024; Rao et al., 2022; Johnson et al., 2023). Research has demonstrated clear molecular mechanisms linking ferroptosis to cognitive dysfunction (Bazinet and Layé, 2014; Bayir et al., 2006; Yoo et al., 2012). At the molecular level, GPX4 deficiency leads to increased lipid peroxidation and glutathione depletion, triggering ferroptosis (Bayir et al., 2002; Friedmann Angeli et al., 2014). This cell death mode particularly affects hippocampal neurons, resulting in synaptic dysfunction manifested by abnormal expression of presynaptic marker SYN and postsynaptic scaffold protein PSD95 (Hambright et al., 2017; Chen et al., 2015). Additionally, ferroptotic neurons activate neuroinflammation through the release of damage-associated molecular patterns (DAMPs) and lipid metabolites (Bayir et al., 2002; Friedmann Angeli et al., 2014). These pathological changes ultimately lead to cognitive impairment, particularly in spatial learning and memory (Chen et al., 2015). Notably, GPX4 overexpression or the use of ferroptosis inhibitors can significantly ameliorate these pathological alterations (Hambright et al., 2017). Additionally, tau pathology is a common pathological basis for neurodegenerative processes that develop after TBI. Meanwhile, ferroptosis can occur in various areas of the central nervous system, including the cerebral cortex, hippocampus, striatum, and spinal cord (Yao et al., 2021). Its role in causing secondary damage after spinal cord injury has been reported (Ryan et al., 2024). Studies have also found a link between spinal cord injury and cognitive decline (Wecht et al., 2023; Pasipanodya et al., 2021), with evidence indicating that spinal cord injury can affect the control of blood flow to the brain and potentially raise the chances of cognitive problems (Phillips et al., 2013; Wecht et al., 2018).

This discovery regarding the thematic progression of cognitive impairment following TBI aligns with existing literature. TBI resulted in significant neuropathological damage characterized by neuroinflammation, oxidative stress, and progressive neurodegeneration, which collectively contribute to motor and cognitive decline (Nebie et al., 2021). On the other hand, the thematic evolution analysis reflected the research boom in the field of cognitive dysfunction after TBI. In the lower-left quadrant, it is probable that the themes of “pediatric” and “development” are emerging. During adolescence, significant neurodevelopmental and cognitive changes take place, leading to a transition from reactive to proactive forms of cognitive control, such as response inhibition. Pediatric mild TBI can affect cognitive functions immediately after the injury, with mild cognitive issues and clinical symptoms lasting for as long as 4 months after the injury (Mayer et al., 2024; van der Horn et al., 2023).

The primary themes identified were “cognition, rehabilitation, and depression,” with multifaceted symptoms observed following TBI. A closed head injury leading to TBI has been significantly associated with an elevated risk of developing Alzheimer’s disease and chronic traumatic encephalopathy (VanItallie, 2019). While moderate and severe TBI can result in a range of cognitive, emotional, and behavioral consequences, mild TBI is primarily associated with emotional symptoms and mental health conditions like posttraumatic stress disorder and major depressive disorder (Howlett et al., 2022). It has been demonstrated that environmental factors can influence the recovery of cognitive impairment. Changes in diet have been shown to impact cognitive function in older adults undergoing outpatient rehabilitation programs (Uchiyama-Tanaka et al., 2024). Additionally, research has demonstrated that the combination of a diet high in fat and brain injury can modify adipose tissue macrophages and brain microglia, resulting in worsened cognitive impairment (Henry et al., 2024).

The increasing incidence of TBI-related cognitive dysfunction poses a significant public health challenge, highlighting the urgent need for effective strategies in diagnosis, treatment, prevention, and control of cognitive impairments after TBI. This bibliometric analysis provided research directions for scientists in this field from a macroscopic perspective. With the progress of research, neuroimaging technology has become an important tool for diagnosing cognitive dysfunction. Diffusion tensor imaging has emerged as the most commonly used imaging modality for assessing cognitive dysfunction following TBI (Chen et al., 2023). It is worth noting that ferroptosis is associated with the pathophysiology of TBI 24 and might be an important regulatory target for cellular metabolic reprogramming after TBI (Kapralov et al., 2020). Xie et al. demonstrated that intraventricular injection of ferrostatin-1, an inhibitor of ferroptosis, could reduce the severity of lesions after TBI and alleviate some symptoms of cognitive dysfunction (Xie et al., 2019). Inflammation is an essential theme that has received more attention in the field of cognitive dysfunction after TBI. Studies illustrated that inflammation was associated with neurodegenerative changes and cognitive dysfunction for years after TBI (Lu et al., 2023; Johnson et al., 2023). Thus, prevention and control of inflammation may be an effective entry point to reduce cognitive decline after TBI. Additionally, the beneficial effects of rehabilitation on cognitive dysfunction after TBI, including cognitive rehabilitation and neuromodulation techniques, have also been well-documented (Mahncke et al., 2021). In support of this topic, medical policy makers should prioritize the development of epidemiological databases to facilitate future breakthroughs in this field, as well as enhance medical and economic support policies. Enhanced efforts are imperative to advance non-invasive treatment techniques and promote early cognitive-related neurorehabilitation interventions.

## 5 Strengths and limitations

This study represents a comprehensive bibliometric analysis specifically focusing on cognitive dysfunction following TBI. While previous bibliometric studies have examined post-TBI dementia (Sang et al., 2023) or TBI rehabilitation (Liu et al., 2023), our study provides a distinct perspective by analyzing the broader spectrum of cognitive dysfunction after TBI. The 20-year timeframe (2004–2023) of our analysis enables a thorough examination of research trends in this field.

We acknowledge that using only the WoSCC database may have limitations as some relevant documents not included in this database were not considered. However, WoSCC is widely recognized for its comprehensive coverage and high-quality standards in academic publishing. As demonstrated in recent similar bibliometric studies (Simard et al., 2023; Xia et al., 2023), WoSCC remains a reliable source for bibliometric analysis in neuroscience research. We believe our analysis based on WoSCC data can effectively represent the overall landscape and trends in this field. The exclusion of non-English publications is another limitation of our study, as it may have missed some relevant articles published in other languages.

## 6 Conclusion

The occurrence of cognitive dysfunction after TBI has attracted the attention of neurologists worldwide, with an increasing trend in research output. The USA dominated this field. In the future, researchers in this field should pay more attention to ferroptosis, cognitive decline, spinal cord injury, and prognosis as the hotspots of this field, and strengthen the scientific cooperation with the leading countries in this field.

## Data Availability

The original contributions presented in the study are included in the article/Supplementary material, further inquiries can be directed to the corresponding authors.
