# Supplementary material for: Bibliometric analysis of cognitive dysfunction after traumatic brain injury
Source: Front Neurosci. 2025 Feb 25;19:1534735. doi: 10.3389/fnins.2025.1534735 (PMC11893989; doi:10.3389/fnins.2025.1534735)
Supplement: Supplementary file 1 [file Data_Sheet_1.docx]

Supplementary Material

# Supplementary Data

## The search terms for this study:

“Brain Injury, Traumatic” OR “Traumatic Brain Injur*” OR “Trauma*, Brain” OR “Brain Trauma*” OR “Encephalopath*, Traumatic” OR “Traumatic Encephalopath*” OR “Injury, Brain, Traumatic” OR “TBIs” OR “TBI”

“Cognitive Dysfunction*” OR “Dysfunction*, Cognitive” OR “Cognitive Disorder*” OR “Disorder*, Cognitive” OR “Cognitive Impairment*” OR “Impairment*, Cognitive” OR “Mild Cognitive Impairment*” OR “Cognitive Impairment*, Mild” OR “Impairment*, Mild Cognitive” OR “Cognitive Decline*” OR “Decline*, Cognitive” OR “Mental Deterioration*” OR “Deterioration*, Mental”

## The search string for this study:

((TI=(“Brain Injury, Traumatic” OR “Traumatic Brain Injur*” OR “Trauma*, Brain” OR “Brain Trauma*” OR “Encephalopath*, Traumatic” OR “Traumatic Encephalopath*” OR “Injury, Brain, Traumatic” OR “TBIs” OR “TBI”)) OR AB=(“Brain Injury, Traumatic” OR “Traumatic Brain Injur*” OR “Trauma*, Brain” OR “Brain Trauma*” OR “Encephalopath*, Traumatic” OR “Traumatic Encephalopath*” OR “Injury, Brain, Traumatic” OR “TBIs” OR “TBI”)) OR AK=(“Brain Injury, Traumatic” OR “Traumatic Brain Injur*” OR “Trauma*, Brain” OR “Brain Trauma*” OR “Encephalopath*, Traumatic” OR “Traumatic Encephalopath*” OR “Injury, Brain, Traumatic” OR “TBIs” OR “TBI”) AND ((TI=(“Cognitive Dysfunction*” OR “Dysfunction*, Cognitive” OR “Cognitive Disorder*” OR “Disorder*, Cognitive” OR “Cognitive Impairment*” OR “Impairment*, Cognitive” OR “Mild Cognitive Impairment*” OR “Cognitive Impairment*, Mild” OR “Impairment*, Mild Cognitive” OR “Cognitive Decline*” OR “Decline*, Cognitive” OR “Mental Deterioration*” OR “Deterioration*, Mental”)) OR AB=(“Cognitive Dysfunction*” OR “Dysfunction*, Cognitive” OR “Cognitive Disorder*” OR “Disorder*, Cognitive” OR “Cognitive Impairment*” OR “Impairment*, Cognitive” OR “Mild Cognitive Impairment*” OR “Cognitive Impairment*, Mild” OR “Impairment*, Mild Cognitive” OR “Cognitive Decline*” OR “Decline*, Cognitive” OR “Mental Deterioration*” OR “Deterioration*, Mental”)) OR AK=(“Cognitive Dysfunction*” OR “Dysfunction*, Cognitive” OR “Cognitive Disorder*” OR “Disorder*, Cognitive” OR “Cognitive Impairment*” OR “Impairment*, Cognitive” OR “Mild Cognitive Impairment*” OR “Cognitive Impairment*, Mild” OR “Impairment*, Mild Cognitive” OR “Cognitive Decline*” OR “Decline*, Cognitive” OR “Mental Deterioration*” OR “Deterioration*, Mental”)

# Supplementary Figure and Tables

## Supplementary Figure


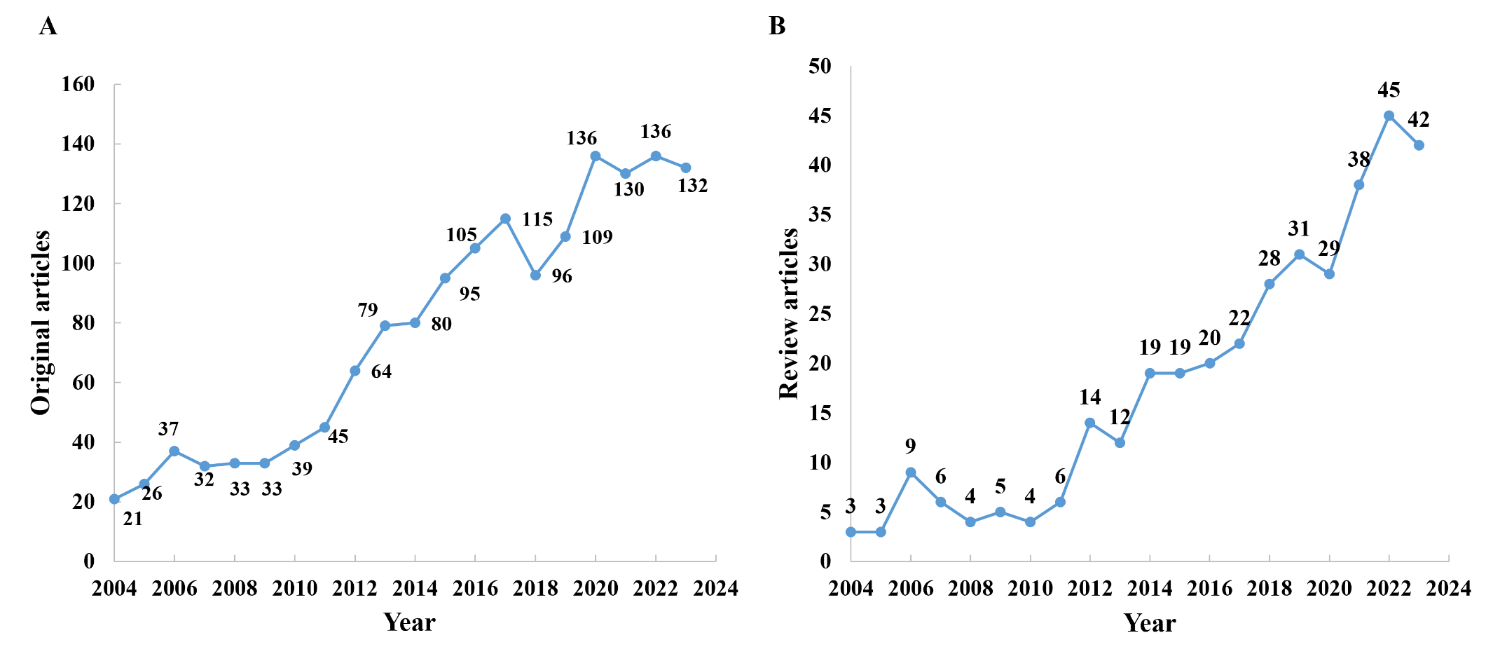


**Supplementary Figure 1**. Annual scientific production and total citation per year of the original articles (A) and review articles (B)

## Supplementary Tables

### Supplementary Table 1 Top 10 most influential journals

| **Original articles** | | |  |  | **Review articles** | | | |  |
| --- | --- | --- | --- | --- | --- | --- | --- | --- | --- |
| **Rank** | **Journal** | **h-index** | **NP** | **TC** | **Rank** | **Journal** | **h-index** | **NP** | **TC** |
| **1** | JOURNAL OF NEUROTRAUMA | 44 | 124 | 5962 | **1** | FRONTIERS IN NEUROLOGY | 12 | 20 | 604 |
| **2** | BRAIN INJURY | 27 | 91 | 2357 | **2** | BRAIN INJURY | 9 | 11 | 378 |
| **3** | JOURNAL OF HEAD TRAUMA REHABILITATION | 22 | 45 | 1271 | **3** | NEUROSCIENCE AND BIOBEHAVIORAL REVIEWS | 8 | 9 | 908 |
| **4** | JOURNAL OF NEUROSCIENCE | 19 | 22 | 1928 | **4** | FRONTIERS IN NEUROSCIENCE | 7 | 8 | 614 |
| **5** | EXPERIMENTAL NEUROLOGY | 17 | 25 | 1419 | **5** | JOURNAL OF ALZHEIMERS DISEASE | 7 | 10 | 229 |
| **6** | JOURNAL OF THE INTERNATIONAL NEUROPSYCHOLOGICAL SOCIETY | 17 | 20 | 873 | **6** | JOURNAL OF NEUROTRAUMA | 6 | 6 | 473 |
| **7** | PLOS ONE | 17 | 31 | 929 | **7** | AGEING RESEARCH REVIEWS | 4 | 4 | 185 |
| **8** | FRONTIERS IN NEUROLOGY | 16 | 42 | 680 | **8** | EXPERIMENTAL NEUROLOGY | 4 | 4 | 900 |
| **9** | CLINICAL NEUROPSYCHOLOGIST | 15 | 24 | 591 | **9** | INTERNATIONAL JOURNAL OF MOLECULAR SCIENCES | 4 | 4 | 46 |
| **10** | BRAIN | 14 | 15 | 3191 | **10** | MOLECULAR NEUROBIOLOGY | 4 | 4 | 263 |

Abbreviations: NP Number of Publications; TC Total Citations.

### Supplementary Table 2 The top 10 affiliations in the number of publications

| **Original articles** | | | **Review articles** | | |
| --- | --- | --- | --- | --- | --- |
| **Rank** | **Affiliation** | **Articles** | **Rank** | **Affiliation** | **Articles** |
| 1 | UNIVERSITY OF CALIFORNIA SYSTEM | 235 | 1 | HARVARD UNIVERSITY | 41 |
| 2 | VETERANS HEALTH ADMINISTRATION (VHA) | 195 | 2 | UNIVERSITY OF TORONTO | 38 |
| 3 | US DEPARTMENT OF VETERANS AFFAIRS | 150 | 3 | VETERANS HEALTH ADMINISTRATION (VHA) | 24 |
| 4 | HARVARD UNIVERSITY | 148 | 4 | TEXAS TECH UNIVERSITY HEALTH SCIENCE CENTER | 21 |
| 5 | UNIVERSITY OF PITTSBURGH | 145 | 5 | HARVARD MEDICAL SCHOOL | 20 |
| 6 | PENNSYLVANIA COMMONWEALTH SYSTEM OF HIGHER EDUCATION (PCSHE) | 134 | 6 | TEXAS TECH UNIVERSITY SYSTEM | 19 |
| 7 | UNIVERSITY OF CALIFORNIA SAN FRANCISCO | 130 | 7 | UNIVERSITY OF PITTSBURGH | 18 |
| 8 | BOSTON UNIVERSITY | 122 | 8 | US DEPARTMENT OF VETERANS AFFAIRS | 18 |
| 9 | UNIVERSITY OF TEXAS SYSTEM | 108 | 9 | BOSTON UNIVERSITY | 16 |
| 10 | UNIVERSITY OF PENNSYLVANIA | 81 | 10 | MASSACHUSETTS GENERAL HOSPITAL | 15 |

### Supplementary Table 3 Top 10 countries in terms of number of publications

| **Original articles** | | | **Review articles** | | |
| --- | --- | --- | --- | --- | --- |
| **Rank** | **Country** | **Articles** | **Rank** | **Country** | **Articles** |
| **1** | USA | 700 | **1** | USA | 140 |
| **2** | CHINA | 221 | **2** | CHINA | 34 |
| **3** | AUSTRALIA | 84 | **3** | CANADA | 27 |
| **4** | UNITED KINGDOM | 67 | **4** | UNITED KINGDOM | 25 |
| **5** | CANADA | 59 | **5** | AUSTRALIA | 22 |
| **6** | JAPAN | 45 | **6** | ITALY | 12 |
| **7** | FRANCE | 37 | **7** | FRANCE | 8 |
| **8** | KOREA | 35 | **8** | NETHERLANDS | 8 |
| **9** | ITALY | 34 | **9** | KOREA | 7 |
| **10** | GERMANY | 24 | **10** | GERMANY | 6 |

### Supplementary Table 4 Top 10 most local cited documents

| **Original articles** | | | | **Review articles** | | | |
| --- | --- | --- | --- | --- | --- | --- | --- |
| **Rank** | **Document** | **LC** | **TC** | **Rank** | **Document** | **LC** | **TC** |
| **1** | MCKEE AC, 2013, BRAIN | 64 | 1415 | **1** | SMITH DH, 2013, NAT REV NEUROL | 13 | 502 |
| **2** | KINNUNEN KM, 2011, BRAIN | 50 | 490 | **2** | JOHNSON VE, 2013, EXP NEUROL | 12 | 782 |
| **3** | RAMLACKHANSINGH AF, 2011, ANN NEUROL | 47 | 696 | **3** | MCINNES K, 2017, PLOS ONE | 9 | 287 |
| **4** | DRAPER K, 2008, NEUROPSYCHOLOGY | 43 | 254 | **4** | BALES JW, 2009, NEUROSCI BIOBEHAV R | 8 | 196 |
| **5** | JOHNSON VE, 2012, BRAIN PATHOL | 42 | 452 | **5** | SIVANANDAM TM, 2012, NEUROSCI BIOBEHAV R | 8 | 221 |
| **6** | DIKMEN SS, 2009, J HEAD TRAUMA REHAB | 41 | 313 | **6** | MANLEY G, 2017, BRIT J SPORT MED | 8 | 394 |
| **7** | OMALU BI, 2005, NEUROSURGERY | 33 | 698 | **7** | WARDEN DL, 2006, J NEUROTRAUM | 7 | 307 |
| **8** | HIMANEN L, 2006, NEUROLOGY | 30 | 160 | **8** | WALKER KR, 2013, FRONT AGING NEUROSCI | 7 | 182 |
| **9** | WITGEN BM, 2005, NEUROSCIENCE | 29 | 164 | **9** | MCKEE AC, 2014, ACTA NEUROPATHOL | 6 | 294 |
| **10** | SCHEID R, 2006, ARCH NEUROL-CHICAGO | 29 | 191 | **10** | STEIN TD, 2014, ALZHEIMERS RES THER | 6 | 161 |

Abbreviations: LC Local citations; TC Total Citations.

### Supplementary Table 5 Top 10 most local cited references

| **Original articles** | | | **Review articles** | | |
| --- | --- | --- | --- | --- | --- |
| **Rank** | **Reference** | **LC** | **Rank** | **Reference** | **LC** |
| 1 | TEASDALE G, 1974, LANCET | 81 | 1 | MCKEE AC, 2013, BRAIN | 42 |
| 2 | MCKEE AC, 2009, J NEUROPATH EXP NEUR | 69 | 2 | MCKEE AC, 2009, J NEUROPATH EXP NEUR | 33 |
| 3 | FOLSTEIN MF, 1975, J PSYCHIAT RES | 67 | 3 | MOHER D, 2015, SYST REV-LONDON | 30 |
| 4 | MCKEE AC, 2013, BRAIN | 64 | 4 | ANONYMOUS, J NEUROSURG ANESTHES | 26 |
| 5 | HOGE CW, 2008, NEW ENGL J MED | 60 | 5 | GUSKIEWICZ KM, 2005, NEUROSURGERY | 25 |
| 6 | LANGLOIS JA, 2006, J HEAD TRAUMA REHAB | 59 | 6 | MCKEE AC, 2010, J NEUROPATH EXP NEUR | 20 |
| 7 | PLASSMAN BL, 2000, NEUROLOGY | 57 | 7 | PLASSMAN BL, 2000, NEUROLOGY | 20 |
| 8 | RABINOWITZ AR, 2014, PSYCHIAT CLIN N AM | 56 | 8 | FLEMINGER S, 2003, J NEUROL NEUROSUR PS | 19 |
| 9 | MAAS AIR, 2017, LANCET NEUROL | 54 | 9 | LEHMAN EJ, 2012, NEUROLOGY | 19 |
| 10 | FAUL MX., 2010, CENTERS FOR DISEASE CONTROL AND PREVENTION, NATIONAL CENTER FOR INJURY PREVENTION AND CONTROL | 51 | 10 | OMALU BI, 2005, NEUROSURGERY | 19 |

Abbreviations: LC Local citations.

### Supplementary Table 6 Top 10 most influential authors

|  | **Original articles** | | | | | **Review articles** | | | |
| --- | --- | --- | --- | --- | --- | --- | --- | --- | --- |
| **Rank** | **Author** | **h-index** | **NP** | **TC** | **Rank** | **Author** | **h-index** | **NP** | **TC** |
| 1 | PICK CG | 16 | 22 | 708 | 1 | IVERSON GL | 6 | 7 | 561 |
| 2 | GREIG NH | 14 | 16 | 678 | 2 | GARDNER AJ | 3 | 4 | 491 |
| 3 | SHARP DJ | 14 | 17 | 3263 | 3 | REDDY PH | 3 | 4 | 113 |
| 4 | RUBOVITCH V | 13 | 15 | 577 | 4 | FILLEY CM | 2 | 4 | 261 |
| 5 | HOFFER BJ | 12 | 14 | 537 | 5 | COLANTONIO A | 3 | 3 | 44 |
| 6 | PONSFORD J | 12 | 16 | 663 | 6 | DIXON CE | 3 | 3 | 362 |
| 7 | TWEEDIE D | 11 | 11 | 530 | 7 | MCCRORY P | 3 | 3 | 482 |
| 8 | COHEN AS | 10 | 11 | 714 | 8 | PASTERNAK JJ | 3 | 3 | 10 |
| 9 | DIXON CE | 10 | 10 | 233 | 9 | SHARP DJ | 3 | 3 | 563 |
| 10 | LEECH R | 10 | 10 | 2411 | 10 | AHNFELDT EP | 2 | 3 | 14 |

Abbreviations: NP Number of Publications; TC Total Citations.
